# Use of Population-Based Compartmental Modeling and Retinol Isotope Dilution to Study Vitamin A Kinetics and Total Body Stores among Ghanaian Women of Reproductive Age

**DOI:** 10.1016/j.cdnut.2024.104484

**Published:** 2024-10-18

**Authors:** Michael H Green, Veronica Lopez-Teros, Joanne Balmer Green, Georg Lietz, Sika M Kumordzie, Anthony Oxley, Ahmed D Fuseini, K Winifred Nyaaba, Emily Becher, Jennie N Davis, K Ryan Wessells, Seth Adu-Afarwuah, Reina Engle-Stone, Marjorie J Haskell

**Affiliations:** 1 Department of Nutritional Sciences, The Pennsylvania State University, University Park, PA, United States; 2 Department of Chemical and Biological Sciences, Universidad de Sonora, Hermosillo, Sonora, Mexico; 3 Human Nutrition Research Centre, Newcastle University, Newcastle Upon Tyne, United Kingdom; 4 Institute for Global Nutrition and Department of Nutrition, University of California, Davis, Davis, CA, United States; 5 Department of Nutrition and Food Science, University of Ghana, Legon, Accra, Ghana

**Keywords:** Ghana, model-based compartmental analysis, retinol isotope dilution, vitamin A status, women of reproductive age

## Abstract

**Background:**

Limited data are available on vitamin A kinetics and total body stores (TBS) in women. Such information can be obtained using compartmental modeling and retinol isotope dilution (RID).

**Objectives:**

Objectives were to apply population-based (“super-subject”) modeling to determine retinol kinetics in nonpregnant Ghanaian women of reproductive age and to use RID to predict TBS in the group and its individuals.

**Methods:**

Women (*n =* 89) ingested a dose of [^2^H_6_]retinyl acetate and blood samples (3/woman) were collected from 6 h to 91 d, with all participants sampled at 14 d, about half at either 21 or 28 d, and each at one other time. Composite data (plasma retinol fraction of dose; FD_p_) were analyzed using Simulation, Analysis and Modeling software to obtain kinetic parameters, TBS, and other state variables as well as model-derived values for the RID composite coefficient *FaS*. The latter were used in the RID equation TBS (μmol) = *FaS* × 1/SA_p_ (where SA_p_ is plasma retinol specific activity) to predict TBS at various times.

**Results:**

Model-predicted TBS was 973 μmol (*n =* 87). Geometric mean RID-predicted TBS was 965, 926, and 1006 μmol at 14, 21, and 28 d, respectively, with wide ranges [for example, 252–3848 μmol on day 14 (*n =* 86)]; TBS predictions were similar at later times. Participants had a mean 2 y of vitamin A in stores and estimated liver vitamin A concentrations in the normal range. Model-predicted vitamin A disposal rate was 1.3 μmol/d and plasma recycling number was 37.

**Conclusions:**

Super-subject modeling provides an estimate of group mean TBS as well as group-specific values for the RID coefficient *FaS*; the latter can be used to confidently predict TBS by RID for individual participants in the group under study or in similar individuals at 14 d or more after isotope ingestion.

**Trial registration number:**

Trial is registered (NCT04632771) at https://clinicaltrials.gov.

## Introduction

Public health professionals continue to be interested in assessing vitamin A status in both groups and individuals in different geographic regions worldwide, as well as in subgroups that are at increased risk of vitamin A deficiency [for example, children and women of reproductive age (WRA)] [1,2]. The retinol isotope dilution (RID) method is currently considered the best technique available for estimating vitamin A total body stores (TBS) in community-based studies, although model-based compartmental analysis has also been used; for a review of both approaches, see [2]. To apply the RID method [for reviews, see Furr et al. [3] and Green and Green [4]], researchers collect a single blood sample at a specified time (for example, 14 d) after subjects ingest a tracer dose of stable isotope-labeled vitamin A and plasma is analyzed for retinol specific activity (SA_p_; labeled tracer/unlabeled tracee). TBS is calculated by applying a prediction equation [for example, see Green et al. [5]] that adjusts SA_p_ [here expressed as fraction of dose in plasma (FD_p_)/μmol retinol in plasma] using correction factors (coefficients) which are related to absorption and retention of the dose and to mixing of the dose with exchangeable vitamin A in stores; values for the coefficients are obtained either from the literature or, preferably, from studies done in similar subjects (real or theoretical). In the case of compartmental modeling, multiple blood samples are collected after isotope administration, and kinetic analysis of plasma tracer response over time provides information on whole-body vitamin A kinetics (for example, utilization rate and retinol recycling among tissues) as well as TBS. Modeling can be applied to data from frequently sampled individuals [6,7] or to a composite (“super-subject”) dataset obtained by sampling each subject at 2–3 times and combining results from all subjects into a single dataset [[8], [9], [10], [11]]. This approach provides estimates of retinol kinetic parameters and TBS for the group and, compared with studies in frequently sampled subjects, it minimizes sampling burden on individuals, may enhance compliance, and may be associated with lower overall costs because the number of analyzed samples would be substantially reduced.

By combining a super-subject modeling study with RID, researchers can more confidently predict TBS in individual subjects by using a model-derived value for the RID coefficient obtained for the time specified for RID group sampling [4]; this value is used with individual subject SA_p_ data at the same time to predict TBS. Researchers can also compare estimates of TBS for the group predicted by RID compared with modeling. Here, we used population-based (super-subject) compartmental modeling and applied the RID technique to study TBS and retinol kinetics among a large group of WRA in the northern region of Ghana, an area in which past surveys documented vitamin A deficiency in ∼30% of children and ∼4% of WRA [12]. By measuring TBS among WRA in this region, the current research laid the groundwork for an ongoing randomized controlled trial (RCT; NCT05178407) that will assess the impact of bouillon cubes fortified with vitamin A, iron, zinc, vitamin B12, and folate on micronutrient status in children 2–5 y of age, nonpregnant, non-lactating WRA 15–49 y of age, and nonpregnant lactating women 4–18 mo postpartum [13]; our study also adds to the limited information available on vitamin A kinetics in women. In addition to the current report, which focuses on vitamin A stores and retinol kinetics, a subsequent paper (Kumordzie et al., manuscript in preparation) will describe indicators and predictors of vitamin A status (including serum retinol, serum retinol-binding protein, TBS, and liver vitamin A concentration) among this group of WRA. Our current modeling results provide group-specific values for the RID composite coefficient so that TBS can be more confidently identified for individual participants as well as for the group; those values will be used to estimate TBS among participants in the RCT. Finally, our design allowed us to compare RID-predicted TBS at various times postdosing, providing new insights into the question of appropriate sampling times for RID and on the potential usefulness of estimating TBS for individuals and the group at multiple times.

## Methods

### Participants

WRA were recruited using a random walk method in Tolon and Kumbungu Districts in the northern region of Ghana. Women were potentially eligible for the study if they were 15–49 y of age, were not pregnant or lactating, did not plan to become pregnant during the next 4 mo, and planned to remain in the study area for the next 4 mo. Written informed consent was obtained from potentially eligible women who were interested in participating in the study and they were scheduled for a morning visit at a mobile clinic for further screening. The study protocol was approved by the Ethical Review Committee of the Ghana Health Services (GHS-ERC 012/07/20) and the Institutional Review Board of the University of California (protocol #1536100); the trial was registered at https://clinicaltrials.gov as NCT04632771 [“Retinol isotope dilution Pilot Study 2 (kinetic study)”].

### Eligibility screening for kinetic study

At the screening visit, the general health status of the women was assessed by questionnaire. Women with severe illness warranting hospital referral, those with chronic medical conditions (for example, malignancy or gastrointestinal disease), and those with congenital anomalies requiring frequent medical attention or potentially interfering with nutritional status were not eligible to participate; in addition, women who were currently participating in a clinical trial were ineligible. Women without any of these conditions were tested for pregnancy (urinary human chorionic gonadotropin) and malaria [Malaria Antigen Test (PF) (for detection of *Plasmodium falciparum*); Oscar Medicare Pvt. Ltd.]; they were excluded if pregnant or positive for malaria. Remaining women were screened for recent morbidity and use of over-the-counter low-dose vitamin A supplements. Women were excluded if they reported fever, vomiting and/or diarrhea during the previous 7 d or use of vitamin A-containing micronutrient supplements more than once/wk in the previous 30 d. Trained staff measured body weight to the nearest 0.1 kg with an electronic scale (Model 874; Seca) and height to the nearest 0.1 cm using a stadiometer (Model 217; Seca). A fingerprick blood sample was collected from remaining women for measurement of both hemoglobin and C-reactive protein (CRP) using a point-of-care device (QuikRead Go, Aidian); women were excluded if they had a hemoglobin concentration <80 g/L and/or a CRP concentration >5 mg/L.

### Vitamin A kinetic study

After an overnight fast, women ingested a capsule containing [^2^H_6_]retinyl acetate [6.84 μmol retinol equivalents (∼2 mg retinol equivalents); Buchem BV] dissolved in sunflower oil; the isotope was certified by the manufacturer to be 99.9% all*-trans* purity and fit for human consumption by Food Chemical Codex testing. The dose was consumed with a glass of water and a snack [bread with a spread composed of palm oil, sugar, hazelnuts, and cocoa (∼150 kcal, ∼7 g fat, and ∼0.8 g fiber)] to facilitate absorption of the dose. During the rest of the day, participants were asked to avoid eating liver or taking a micronutrient supplement that contained vitamin A.

Immediately after isotope dosing (day 0), each woman was randomly assigned to have fasting blood samples collected at three times during the subsequent 91-d super-subject retinol kinetic study. All women were assigned to have a blood sample taken on day 14 postdosing and half were assigned to have a sample collected on day 21, with the other half assigned for day 28; these times were of primary interest for predicting TBS by RID (see subsequent section “RID equation coefficients and prediction of TBS”). A third blood sampling time was randomly assigned to each woman so that there were ≤8 participants per time; the 13 additional times, which were selected using sensitivity analysis [14], were 6, 9, or 12 h or 1, 2, 4, 7, 11, 35, 49, 63, 77, or 91 d. Participants were asked to avoid eating liver or taking a micronutrient supplement during the 24 h before each of their 3 scheduled blood sampling times. A study duration of 91 d was chosen based on results of a pilot study (NCT04632771; “Retinol isotope dilution Pilot Study 1”) done to estimate TBS by RID among different WRA (*n =* 19) from the same communities. That study not only provided an initial estimate of stores but also confirmed that a duration of 91 d was sufficient to ensure accurate definition of the terminal slope of the tracer response curve; additionally, it led us to increase the amount of the tracer dose from 1 to 2 mg retinol equivalents.

### Sample procedures and analyses

At each sample collection time, venous blood (10 mL) was collected from an antecubital vein into evacuated tubes (red-top serum tubes containing clot activator; BD Vacutainer). For samples collected on days 21 or 28, duplicate aliquots (50 μL) of venous blood were taken to determine hematocrit by centrifugation (GCH-24 Hematocrit Centrifuge, Globe Scientific, Inc.) at 15,800 × g for 3 min. Evacuated tubes were wrapped in aluminum foil to protect the sample from light and then immediately placed in coolers with cold packs or in a portable refrigerator (4°C); blood was allowed to clot for 30–60 min and then centrifuged within 8 h. Serum was aliquoted into cryovials and stored at −80°C at the field office in Tolon until samples on cold packs were shipped by air to the University of Ghana-Legon, in Accra, where they were stored at −80°C. Thereafter, serum samples were shipped with dry ice to Newcastle University for analysis of concentrations of [^2^H_6_]retinol and [^12^C]retinol by LC/MS/MS [15].

### Kinetic analysis

**FIGURE 1:**
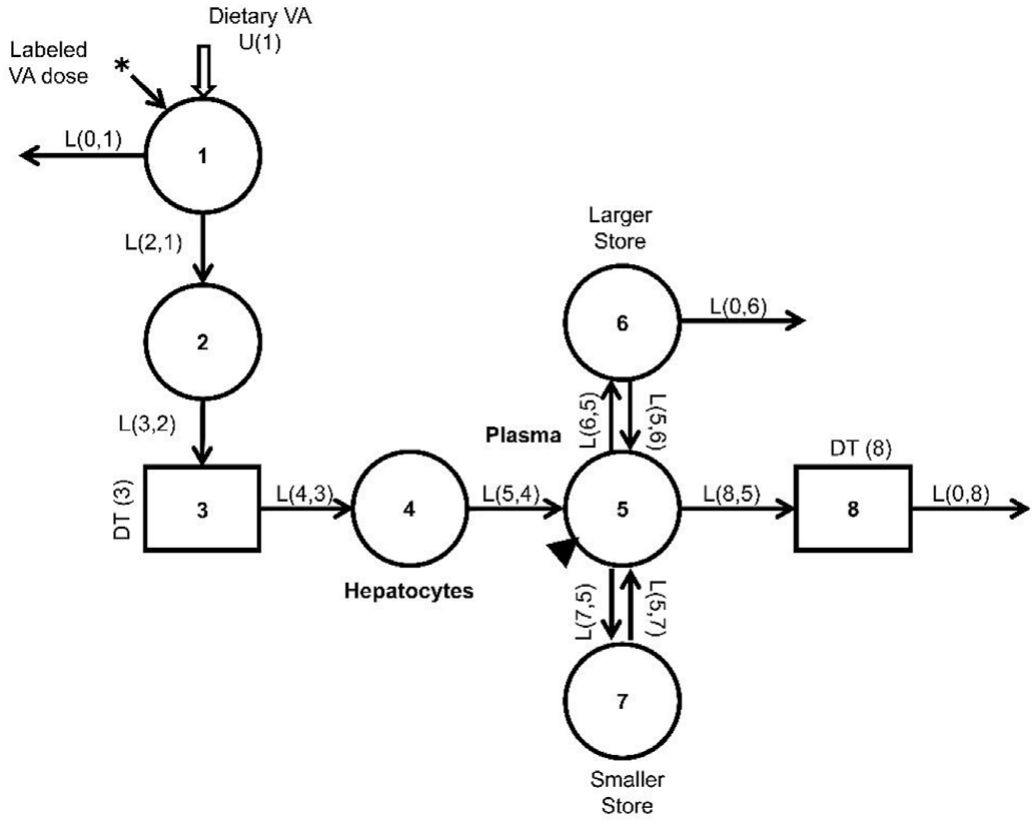
Proposed compartmental model for whole-body vitamin A metabolism in women of reproductive age [adapted from Green and Green [20] ]. Circles represent compartments; the rectangles are delay elements; interconnectivities between compartments (arrows) are fractional transfer coefficients [L(I,J)s, or the fraction of retinol in compartment J transferred to compartment I each day] and delay times [DT(I)s, or days spent in delay element I]. Compartment 1 is the site of introduction of the ingested vitamin A tracer (∗) and dietary vitamin A [U(1)]. Compartments 1–3 represent digestion, absorption, and chylomicron processing until uptake of chylomicron remnant retinyl esters by hepatocytes (compartment 4), with subsequent secretion of retinol bound to retinol-binding protein into plasma compartment 5; compartment 5 is the site of sampling (triangle). Component 8 allows for irreversible uptake of plasma retinol by tissues from which retinol does not recycle. Retinol in plasma can also exchange with vitamin A in 2 extravascular pools (a larger compartment 6 and a smaller compartment 7, which together comprise vitamin A total body stores), with compartments 6 and 8 the sites of irreversible loss from the system. VA, vitamin A.

Fraction of the [^2^H_6_]retinol dose in plasma (FD_p_) was calculated for each subject at each sampling time from 6 h to 91 d after isotope administration as {[^2^H_6_]retinol (μmol/L) × estimated plasma volume (L)} / dose (μmol), where plasma volume was estimated using the regression equation of Nadler et al. [16] and hematocrit, as described by Ford et al. [17]. The geometric mean FD_p_ for all subjects sampled at each time was computed to obtain a composite (super-subject) dataset. The super-subject dataset was analyzed by model-based compartmental analysis using WinSAAM, the Windows version of the Simulation, Analysis and Modeling software [www.winsaam.org; [14,18,19] ], in light of a previously published 8-compartment model [20] for whole-body vitamin A kinetics in adults (Figure 1). In this model, compartment 1 is the site of introduction of ingested tracer and dietary vitamin A. Components 1–3 represent vitamin A digestion and absorption as well as production and metabolism of retinyl ester-containing chylomicrons. Resulting chylomicron remnants are taken up by hepatocytes (compartment 4), with subsequent secretion of retinol bound to retinol-binding protein into plasma compartment 5. From compartment 5, retinol is either delivered to delay component 8, which allows for irreversible uptake and utilization of plasma retinol by tissues from which it does not recycle, or it can exchange with vitamin A in 2 extravascular pools (a larger compartment 6 and a smaller compartment 7, which together comprise TBS); compartments 6 and 8 are the sites of irreversible loss from the system. For modeling, we assumed that subjects were in vitamin A balance during the 91-d study.

Geometric mean data for FD_p_ over time were fit to the model using WinSAAM by iteratively adjusting values for the seven adjustable fractional transfer coefficients [L(I,J)s, or the fraction of retinol in compartment J transferred to compartment I each day] and the time (d) retinol spent in delay component 3 [DT(3)]. In view of the number of subjects sampled at each time and based on previous modeling experience, we assigned a weight [fractional standard deviation (FSD)] of 0.02 to the 14-d data when results were available for 86 women, an FSD of 0.05 to data for days 21 and 28 when data were available for about half of the participants at each time (*n =* 40 and 42, respectively), and larger FSDs (0.5 for the 7 and 35 d data when *n =* 4 and 0.1 at the 11 other times when there were 5–8 subjects). As in previous retinol kinetic studies [6,21], we assumed a vitamin A absorption efficiency of 75%. We also specified, as in Ford et al. [11], that R(8,5) = R(0,6) [that is, 50% of the loss from the system was from compartment 6 (Figure 1) and 50% was from component 8]. This partitioning is derived from data on sites of tracer loss from the system over time in rats (unpublished observations). That work indicated that early loss was most likely from tissues which obtained vitamin A directly from plasma (that is, was likely related to functional use of the vitamin), whereas loss which occurred later was from stores (that is, was more likely degradative). Final values for the adjustable parameters and their statistical uncertainties (FSDs) were obtained by nonlinear regression analysis using WinSAAM. Then, M(5), the geometric mean mass (μmol) of retinol in plasma (compartment 5), was calculated from each individual’s day 14 plasma retinol concentration (μmol/L) × their estimated plasma volume (L). The calculated value for M(5) was used in a steady state solution to the model, which provided estimates for the mass of retinol in other compartments (including TBS), as well as rates of transfer of vitamin A [R(I,J)s, where R(I,J) = L(I,J) × M(J)] and other parameters of interest; see **Supplemental WinSAAM Deck** and Cifelli et al. [22].

### RID equation coefficients and prediction of TBS

In addition to estimating group mean TBS by modeling, we also used model-derived information to obtain time-specific values for the RID composite coefficient *FaS* for use in RID Equation 1 [5] so that we could predict TBS from measured SA_p_:

[1] TBS (μmol) = *Fa* × *S* × (1/SA_p_)

where *Fa*, the fraction of the orally administered stable isotope dose in stores at time *t*, is calculated as the sum of the FD in the 2 storage compartments that comprise TBS [compartments 6 and 7 (Figure 1)] and coefficient *S* was simulated as SA_p_/SA_s_, with SA_p_ equal to the FD_p_ at time *t* divided by the plasma total retinol pool size (μmol) and SA_s_ equal to *Fa* divided by the total amount of vitamin A in the 2 storage compartments. We simulated values for *FaS* (see Supplemental WinSAAM Deck) over time for this group of women. Then we applied Equation 1, using the values for *FaS* and SA_p_ on postdosing days 14, 21, 28, 35, 49, 63, 77, and 91 to calculate TBS for individual subjects and the group. We collected a blood sample from all subjects on day 14 because that is a time used in several previous RID studies done in community settings [[23], [24], [25]]. We also obtained samples from half of the participants at day 21, as suggested by Furr et al. [2], or at day 28 for comparison with the 14-d RID mean. These later times have recently been shown to provide more accurate predictions of TBS in theoretical subjects [20] because they are times at which the coefficient of variation as a percent (CV%) for *FaS* is lowest; they are also associated with improved identifiability of model parameters. In addition, group mean RID predictions at other times were also compared with the primary results to explore the feasibility of using a variety of sampling times in the field, in case (for example) a subject missed an assigned time for blood sampling. Finally, liver vitamin A concentrations were estimated assuming that 80% of total body vitamin A is found in the liver [26] and that liver weight is 2.4% of body weight [27].

### Data manipulation and statistics

**FIGURE 2:**
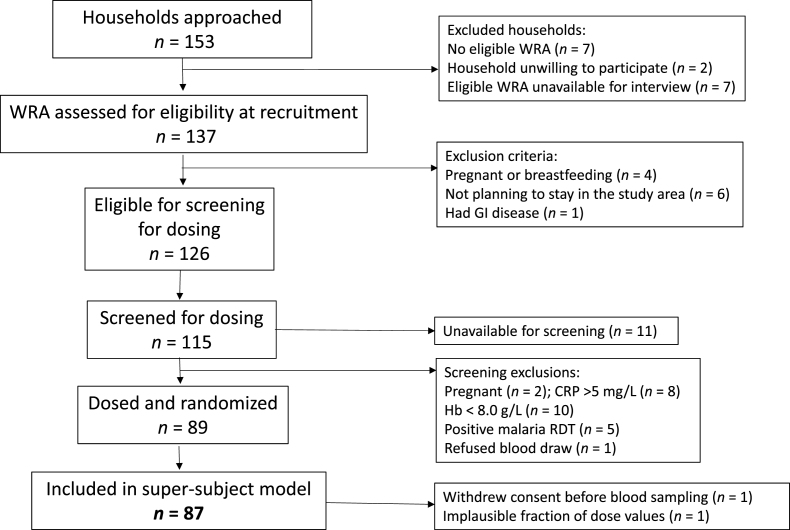
Flowchart summarizing recruitment, assessment, exclusion criteria, and selection of participating WRA. For each sampling time, the following list shows the number of women randomly assigned for blood sampling followed by the number whose FD_p_ results were included in the super-subject model: 6 h (7, 7); 9 h (7, 7); 12 h (7, 7); 1 d (6, 6); 2 d (7, 6); 4 d (6, 6); 7 d (5, 4); 11 d (6, 6); 14 d (89, 86); 21 d (43, 40); 28 d (46, 42); 35 d (5, 4); 49 d (7, 7); 63 d (6, 5); 77 d (10, 7); 91 d (10, 8). Reasons for missing a blood sampling were: unavailable (*n =* 7), dropped out (*n =* 1), reported pregnancy (*n =* 1). CRP, C-reactive protein; GI, gastrointestinal; Hb, hemoglobin; RDT, rapid diagnostic test; WRA, women of reproductive age.

Data are presented as mean ± SD or as geometric mean with range. Data were managed in Microsoft Excel which was also used to calculate Spearman rank correlations, with *P* < 0.05 considered significant. GraphPad Prism 10.0 for Windows (GraphPad Software) was used to create graphics, except that Microsoft PowerPoint was used to create Figures 1 and 2.

## Results

### Participants

Figure 2 summarizes the number of women recruited and selected for the study. Of the 89 women who met the eligibility criteria for inclusion in the kinetic study, 1 withdrew consent after ingestion of the oral dose of [^2^H_6_]retinyl acetate before any blood samples were obtained; samples were collected for analysis from the remaining 88 subjects but data for 1 woman were excluded because the analytical results and consequent calculation of FD_p_ for all 3 of her samples were implausibly low, presumably due to incomplete ingestion of the tracer dose. The dataset for modeling included results for 87 participants; 75 of the participants were sampled at their 3 assigned times, 11 were sampled at 2 times, and 1 at only 1 time. The sample size for RID predictions of TBS on day 14 was 86 because blood was not obtained from 1 person at that time and data were excluded for the above-mentioned subject with implausibly low results. Participant characteristics are shown in Table 1; additional details will be presented separately (Kumordzie et al., manuscript in preparation).

**TABLE 1:** Participant characteristics at enrollment (*n =* 87)^1^.

Characteristic	Mean ± SD	Min, Max
Age (y)	31 ± 8.6	(15, 46)
Weight (kg)	57 ± 9.6	(37, 82)
Height (m)	1.60 ± 0.059	(1.46, 1.74)
BMI (kg/m^2^)	22 ± 4.0	(15, 34)
Hemoglobin (g/L)	127 ± 13	(88, 157)

Abbreviations: Max, maximum; Min; minimum; WRA, women of reproductive age.

1 Shown are mean ± SD, and min and max values, for characteristics of enrolled WRA. Note that 89 women were enrolled but 2 of them were not part of the super-subject dataset: one woman withdrew consent before blood sampling and a second one had implausible values for fraction of dose in plasma.

### Vitamin A kinetics

**FIGURE 3:**
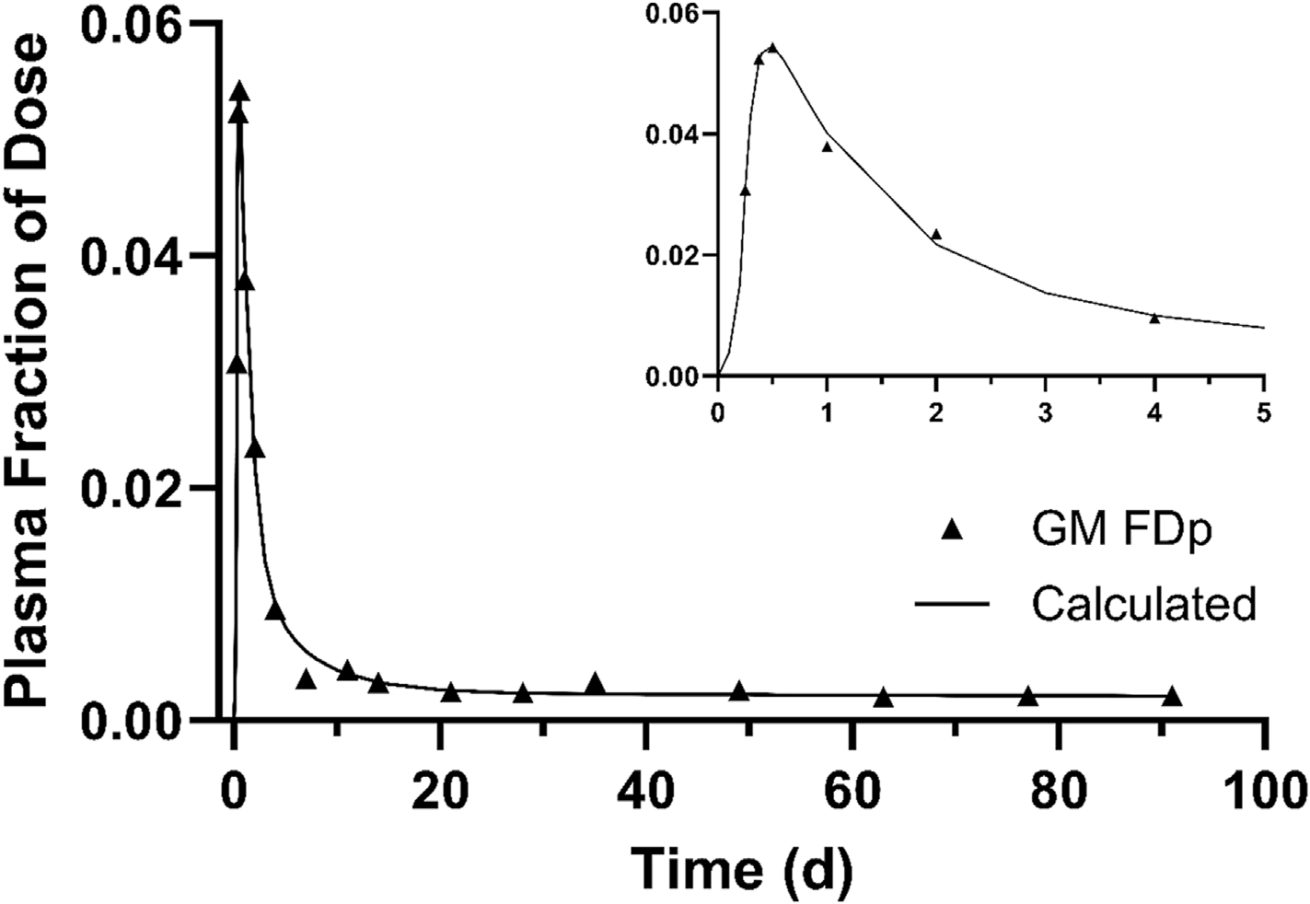
Geometric mean observed and model-calculated plasma retinol fraction of dose among Ghanaian women (15–46-y-old) after ingestion of [^2^H_6_]retinyl acetate. The inset shows the data ≤4 d postdosing on an expanded scale. The composite dataset includes results for all included subjects (*n =* 87); 86 participants were sampled on 14 d postdosing, blood was collected from approximately half of them at days 21 and 28 (*n =* 40 and 42, respectively), and from 4 to 8 subjects at the other sampling times; note that data for 1 woman was not included in the composite dataset for modeling because FD_p_ values were implausible. The model is shown in Figure 1. FD_p_, fraction of dose in plasma retinol; GM, geometric mean.

The composite (super-subject) observed and model-predicted FD_p_ from 6 h to 91 d after ingestion of [^2^H_6_]retinyl acetate in this group of Ghanaian WRA is presented in Figure 3; data for individuals sampled at each time are shown in Supplemental Figure 1. As has been found in other studies, FD_p_ peaked at ∼12 h postdosing; then it fell and showed a bend that is indicative of mixing of the tracer with vitamin A in extravascular pools; at ∼40 d, the curve entered a terminal slope, reflecting the system fractional catabolic rate and indicating that the tracer had mixed completely with exchangeable body vitamin A. At 91 d postdosing, model-predicted FD_p_ was 64% of the day 14 value.

Table 2 shows the final retinol kinetic parameters (fractional transfer coefficients and delay times) and their FSDs derived from modeling the composite dataset for FD_p_. The adjustable model parameters were well identified, with FSDs ranging from 0.03 to 0.20. When the calculated value for mass of retinol in plasma [M(5) = 3.36 μmol] was included in a steady state solution, model-predicted TBS (compartments 6 and 7; Figure 1) was 973 μmol; this represents ∼2-y worth of vitamin A in stores, calculated as TBS/disposal rate (DR), using the model-predicted DR of 1.3 μmol/d (Table 2). Other model predictions included a transit time for retinol in plasma of 2.3 h and a residence time of 2.6 d, where transit time is the time an average retinol molecule remains in a compartment before leaving reversibly or irreversibly and residence time is the total time it spends in plasma before leaving irreversibly [22]; residence time reflects the recycling of retinol to plasma. In addition, the model predicted that a given retinol molecule recycled to plasma 37 times before irreversibly leaving plasma and that it took 30 d for retinol to recycle to plasma after having left for the storage compartments; the system FCR was 0.133%/d and for the stores, this would represent a t_1/2_ of 376 d (because t_1/2_ = 50% / 0.133%/d). Finally, the model-predicted value for dietary vitamin A intake [U(1); Figure 1] was 1.73 μmol/d (496 μg retinol activity equivalents/d), which is 71% of the RDA for nonpregnant, nonlactating adolescent or adult WRA and essentially 100% of the EAR for adolescent and adult WRA [28].

**TABLE 2:** Retinol kinetic parameters and state variables for a group of Ghanaian women of reproductive age^1^.

Parameter	Value	FSD
L(2,1) (d^-1^)	30	—
L(0,1) (d^-1^)	9.99	—
L(3,2) (d^-1^)	30	—
L(5,2) (d^-1^)	0.3	—
L(4,3) (d^-1^)	1	—
L(5,4) (d^-1^)	0.946414	0.106378
L(6,5) (d^-1^)	5.23994	0.031272
L(5,6) (d^-1^)	0.0184312	0.049916
L(7,5) (d^-1^)	4.73927	0.189522
L(5,7) (d^-1^)	0.302567	0.200981
L(0,6) (d^-1^)	0.00070779	0.071801
L(8,5) (d^-1^)	0.19378	—
L(0,8) (d^-1^)	1	—
DT(3) (d)	0.129438	0.076771
DT(8) (d)	0.052	—
State variables		
M(5) (μmol)	3.362	—
M(6) (μmol)	920.458	—
M(7) (μmol)	52.6608	—
TBS (μmol)	973	—
R(8,5) (μmol/d)	0.651488	—
R(0,6) (μmol/d)	0.651488	—
DR (μmol/d)	1.303	—
U(1) (μmol/d)	1.73687	—

Abbreviations: FSD, fractional standard deviation; TBS, vitamin A total body stores.

1 Shown are retinol kinetic parameters (along with FSDs for adjustable parameters) and state variables derived from modeling a composite (super-subject) dataset obtained from a group of Ghanaian women of reproductive age (*n =* 87). Kinetic parameters listed are fractional transfer coefficients [L(I,J)s, or the fraction of retinol in compartment J transferred to compartment I each day except that, following WinSAAM convention, output from a delay element is set at 1] and delay times [DT(I)s, or the time (d) spent in delay component I]; state variables are compartment vitamin A masses [M(I)], including TBS (compartments 6 + 7), transfer rates [R(I,J)s, or the mass of retinol transferred from compartment J to compartment I each day], vitamin A disposal rate (DR, or the amount of retinol irreversibly transferred out of the system from compartments 6 and 8 each day), and dietary vitamin A intake [U(1)]; vitamin A absorption efficiency was set at 75%. The model is shown in Figure 1.

### *FaS* and RID-predicted TBS

The model-derived values for the RID composite coefficient *FaS*, as well as its components *Fa* and *S,* at 14, 21, and 28 d postdosing, as well as at other selected times, are shown in Table 3. The value for *FaS* was 0.983 at 14 d; it was lower at 21 and 28 d (0.763 and 0.691, respectively), and lower still (0.610) by 91 d.

**TABLE 3:** RID coefficients (*FaS, Fa*, and *S)* and RID-predicted TBS among Ghanaian women of reproductive age at 8 times after tracer administration^1^.

Time (d)	*FaS*	*Fa*	*S*	TBS (μmol)			*n*
				GM	Min	Max	
14	0.983	0.720	1.33	965	252	3848	86
21	0.763	0.714	1.05	926	301	3598	40
28	0.691	0.708	0.968	1006	303	2338	42
35	0.665	0.701	0.945	923	555	1286	4
49	0.645	0.689	0.937	901	326	1828	7
63	0.633	0.676	0.936	866	358	2088	5
77	0.621	0.664	0.936	942	540	1370	7
91	0.610	0.653	0.936	935	463	3875	8
—	—	—	Mean	933	—	—	—

Abbreviations: FD_p_, fraction of tracer dose in plasma retinol; GM, geometric mean; Max, maximum; Min, minimum; RID, retinol isotope dilution; TBS, vitamin A total body stores.

1 Shown are values for the RID composite coefficient *FaS,* as well as coefficient components *Fa* and *S*, derived by modeling a composite FD_p_ dataset obtained by super-subject blood sampling among a group of Ghanaian women of reproductive age at various times over 91 d after ingestion of a tracer dose of [^2^H_6_]retinyl acetate. Also shown are GM (with Min and Max) values for TBS (μmol) predicted by RID Equation 1 (see Methods) for participating women sampled at the various times. The model-predicted TBS for the group was 973 μmol. The model is shown in Figure 1.

**FIGURE 4:**
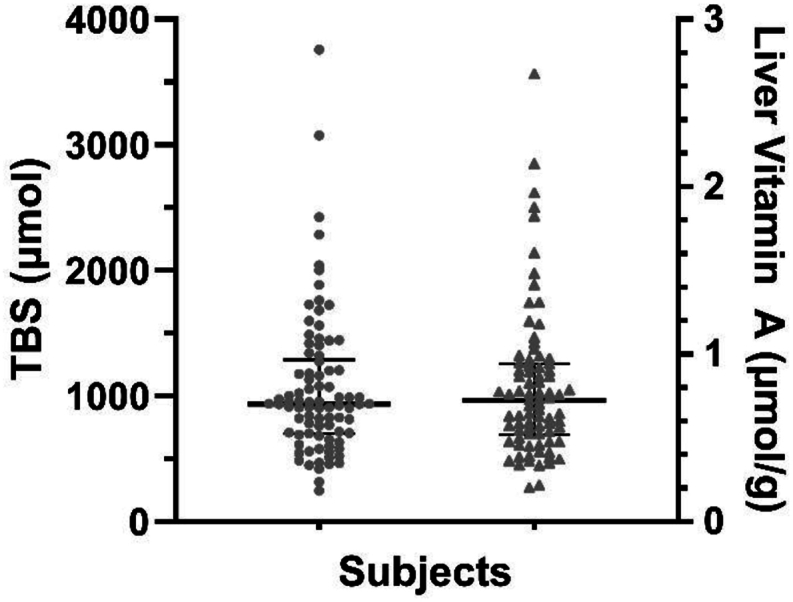
RID-predicted TBS (left) and estimated liver vitamin A concentrations (right) among Ghanaian women of reproductive age 14 d after ingestion of a tracer dose of [^2^H_6_]retinyl acetate. Values for TBS (*n =* 86; μmol) were predicted by RID Equation 1 (see Methods) using a model-derived value of 0.983 for the composite coefficient *FaS* on day 14 (Table 3), along with individual subject data for retinol specific activity in plasma at the same time. Liver vitamin A concentrations (*n =* 86; μmol/g) were estimated using RID-predicted TBS and assuming that 80% of TBS is found in the liver [26] and that liver weight is 2.4% of body weight [27]. RID, retinol isotope dilution; TBS, vitamin A total body stores.

Using the geometric mean value for *FaS* on day 14 in RID Equation 1, along with individual subject values for SA_p_ at the same time, we calculated TBS for each participant (*n =* 86; Figure 4). As summarized in Table 3, the day 14 geometric mean TBS was 965 μmol (range, 252–3848 μmol), essentially the same as the model-predicted value (973 μmol). The values for individual subjects were positively skewed (Figure 4), with TBS ranging from 750–1500 μmol for 58% of the women. Also shown in Table 3 are geometric mean (with minimum and maximum) values for TBS predicted using RID Equation 1 at 7 sampling times after 14 d. Over all times, the mean (933 μmol; range, 886–1006 μmol) was similar to the day 14 value. There was a high rank correlation between RID-predicted TBS on day 14 when all subjects were sampled and values predicted on the two days when half of the group was sampled (Spearman rank correlation *R*^2^ = 0.91 for day 14 compared with 21 and 0.83 for day 14 compared with 28; *P* < 0.001 for both). In addition, when we compared RID-predicted TBS for the 29 subjects whose third blood sample was obtained after day 28 with their day 14 and 21 or 28 prediction, we observed that the estimates of TBS were generally consistent at the 3 times, with a mean CV% of 10.

We also used the RID results to estimate liver vitamin A concentrations, an index sometimes used [29,30] to indicate vitamin A status. On day 14 postdosing, geometric mean liver vitamin A concentration (Figure 4 and Table 4) was 0.718 μmol/g (range, 0.202–2.73 μmol/g). On the basis of the criterion [29,30] that a concentration of ≤0.07 μmol/g indicates vitamin A deficiency, none of the study participants were deficient, 69 (80%) had estimated liver vitamin A concentrations in the normal range (0.1–1.0 μmol/g), and 17 (20%) had high but not toxic concentrations (>1 and <3 μmol/g, respectively).

**TABLE 4:** Estimated mean liver vitamin A concentrations (μmol/g) among Ghanaian women of reproductive age at 8 times after tracer administration^1^.

Time (d)	14	21	28	35	49	63	77	91
*N*	86	40	42	4	7	5	7	8
GM	0.718	0.674	0.763	0.667	0.614	0.668	0.678	0.688
Min	0.202	0.190	0.234	0.441	0.206	0.277	0.391	0.407
Max	2.73	2.50	2.16	0.858	1.16	1.67	1.05	2.69

Abbreviations: GM, geometric mean; Max, maximum; Min, minimum; RID, retinol isotope dilution; TBS, vitamin A total body stores.

1 Shown are GM (Min, Max) liver vitamin A concentrations (μmol/g) at 8 times after women ingested a tracer dose of [^2^H_6_]retinyl acetate. Vitamin A concentrations were calculated based on TBS values predicted by RID Equation 1 (see Methods), using values for the composite coefficient *FaS* (Table 3) derived by modeling, along with individual subject data for retinol specific activity in plasma at the same time, and assuming that 80% of TBS is found in the liver [26] and that liver weight is 2.4% of body weight [27].

## Discussion

As originally designed, the focus of the experiment described here was to determine the typical range for TBS among WRA in northern Ghana and to estimate coefficients for the RID equation; this baseline information was needed so that the research team would be able to assess the impact of multiple-micronutrient fortified bouillon on TBS in a subsequent RCT [13]. TBS results [both those predicted by compartmental modeling (973 μmol) and the day 14 RID (965 μmol)] indicate that, with a mean value of 2-y worth of vitamin A in stores, vitamin A status was very adequate, albeit quite variable, among these women.

In addition to results on vitamin A status, several other aspects of this study are worth highlighting. First, because whole-body retinol metabolism has not been extensively studied in women, our results add to the available literature. Furthermore, the kinetic parameters determined for this group of WRA could be adjusted and used to establish parameters for any number of theoretical subjects with adequate TBS; those hypothetical individuals could be used to study other questions related to vitamin A metabolism [31] such as the impact of a vitamin A intervention on retinol kinetics and TBS. For the current group of WRA, the composite tracer response curve (Figure 3) shows features indicative of tracer absorption, distribution, and metabolism that have been previously described for both rats and humans [for example, see Furr et al. [3] ]; the length of this study (91 d after isotope administration) was sufficient to accurately define the terminal slope of the curve and thus the system fractional catabolic rate for exchangeable vitamin A (0.13 %/d). Model-predicted vitamin A DR in these women was ∼1.3 μmol/d (Table 2), which is lower than that reported for older United States men and women [2.2 μmol/d [32] ] who also had relatively high stores and lower than for healthy United States WRA estimated at 133 d postdosing [2.1 μmol/d [9] ]. The high recycling number for current subjects (37 times) provides confirmation that vitamin A metabolism is a high-response system, as suggested previously by Green et al. [33] based on modeling studies in rats.

Second, as stated earlier, the value for TBS predicted by modeling the composite dataset for FD_p_ (973 μmol) was very close to the geometric mean value predicted by RID at 14 d postdosing (965 μmol; *n =* 86; Table 3). Our findings that RID and super-subject compartmental modeling of vitamin A kinetic data from a large community-based study provide similar estimates of TBS confirm observations in other modeling studies in children [8,34,35] and young adults [7], as well as the outcome predicted by Green’s group for theoretical adults [4].

The limited data currently available in children [8,11] and theoretical subjects [20] indicate that the RID equation composite coefficient *FaS* varies among groups; this study provides such information for WRA (Table 3). Although it is not surprising that *FaS* is group specific, this result underscores the value of including a super-subject modeling component, as we did here, when RID will be used to estimate TBS in a group or individuals. Specifically, the super-subject approach provides not only an estimate of TBS using a method other than RID but, importantly, it provides group-specific values for the coefficients *Fa* and *S* over time (Table 3). As noted in Methods, *Fa* represents the FD_p_ in stores over time (Table 3). *Fa* rises to a maximum and then very slowly decreases based on the system FCR. *S*, the ratio of vitamin A specific activity in plasma to that in stores, is initially very high but then falls as vitamin A specific activity rises in stores. After several weeks to a month, when the tracer has thoroughly mixed with tracee, *S* reaches a plateau and remains there. Here, we used the model-generated values for *Fa* and *S* in Equation 1 to confidently predict TBS for both the group (Table 3) and for individual participants (Figure 4) at various times from 14 to 91 d postdosing. Note that, although RID has been assumed to be best applied to assess vitamin A status in groups rather than its individuals [2], using group-specific values for *FaS*, as opposed to generic values, removes this limitation, assuming that the RID is done at appropriate times (for example, 14–28 d postdosing). That is, using model-derived values for *FaS* to predict TBS for individuals in a study group, as was done here and in Engle-Stone et al. [36], or in a similar group of subjects, such as the RCT mentioned earlier, will provide estimates of stores that are as accurate as one could currently obtain, given the state-of-the-art for the RID method. Having reliable individual values for TBS will enable researchers to examine relationships between vitamin A stores and other variables (such as vitamin A intake, morbidity, inflammatory status, etc.).

This is also the first community-based study in which TBS predicted by RID at various times postdosing were intentionally compared. We found good agreement between predictions at 14, 21, and 28 d (Table 3); predictions at later times, when small numbers of subjects were evaluated, were also similar. Sampling at 14 d postdosing has been previously used for RID in community studies [23,37,38] and the 2 later times were recommended based on work in theoretical subjects [20], which indicated both that the most accurate RID predictions of individual subject TBS were obtained at 14 or more days postdosing and that, for adults with high vitamin A stores, sampling at 21 or 28 d postdosing, when the CV% for *FaS* was lowest, would provide the most accurate predictions. This is because, if the CV% for *FaS* is low, then the variance in TBS is predicted by the measured variable (SA_p_) for each individual.

Also of interest, we found that RID predictions in subjects whose third sample was collected after 28 d (*n =* 29) were quite close within individuals. Similar results were presented by Engle-Stone et al. [36] who reported that RID-predicted TBS in Filipino children sampled at 4 d postdosing was highly correlated with values calculated based on analysis of samples obtained at 16–28 d. Taken together, these results suggest that, when combining a super-subject approach with RID as described here, researchers may have more flexibility for blood sampling than previously assumed. For example, if a subject was unable to have a blood sample collected at the time specified for RID in all subjects, that sample could be obtained (for example) the day before or after and those results could be confidently included with others obtained on the specified day.

In conclusion, on the basis of both model-predicted and 14-d RID-predicted TBS (973 and 965 μmol, respectively), as well as estimated liver vitamin A concentrations (Table 4), the Ghanaian women studied here had very adequate vitamin A stores. However, individual subject values for TBS varied over a wide range (252–3848 μmol). The protocol used here (super-subject modeling of a composite dataset combined with RID to obtain individual values for TBS with a high degree of confidence) provided several new insights that expand the flexibility of the RID method. We suggest that the approach we describe here may be an optimal and feasible one for future community-based studies on vitamin A status, especially when researchers are interested in individual subject TBS.

## Author contributions

The authors’ responsibilities were as follows – MJH, GL, MHG, RE-S: designed the study; SMK, ADF, KWN, AO, KRW, EB, JND, SA-A: conducted the study; MHG, VL-T: did the modeling and interpreted the results; MHG, JBG, MJH: wrote the first draft of the manuscript; MHG, MJH: have primary responsibility for final content; and all authors: read and agreed to the final version of the manuscript.

## Conflict of interest

RE-S is an Editorial Board Member for Current Developments in Nutrition and played no role in the Journal's evaluation of the manuscript. The other authors report no conflicts of interest.

## Funding

This work was supported in whole or in part by a grant from Helen Keller International (66504-UCD-01) through the Bill & Melinda Gates Foundation (INV007916). Neither agency had any involvement in study design, data analysis or interpretation, writing of the manuscript, or the decision to submit for publication. The content is solely the responsibility of the authors and does not necessarily represent the official positions of the Bill & Melinda Gates Foundation.

## Data availability

Data are available upon request from the corresponding authors.
