# Supplementary material for: Use of Population-Based Compartmental Modeling and Retinol Isotope Dilution to Study Vitamin A Kinetics and Total Body Stores among Ghanaian Women of Reproductive Age
Source: Curr Dev Nutr. 2024 Oct 18;8(11):104484. doi: 10.1016/j.cdnut.2024.104484 (PMC11616041; doi:10.1016/j.cdnut.2024.104484)
Supplement: multimedia component 1 [file mmc1.docx]

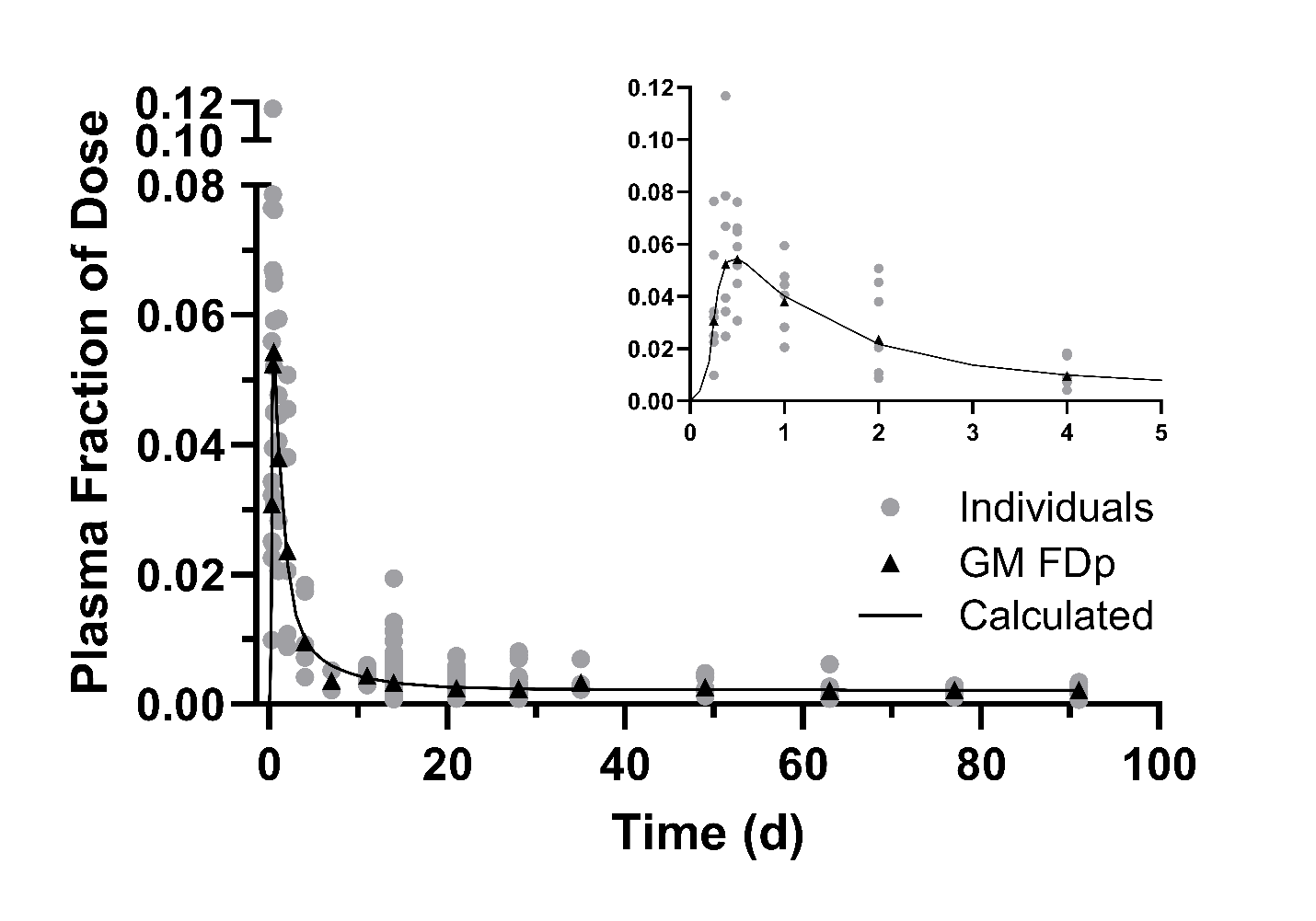


**Supplemental Figure 1** Individual observed data for plasma retinol fraction of dose versus time after ingestion of [^2^H_6_]retinyl acetate in Ghanaian women of reproductive age, GM values at each time, and model-calculated curve for the GM data; the inset shows values for the first 4 d postdosing on an expanded scale. The number of women sampled at each time was 6 h (7); 9 h (7); 12 h (7); 1 d (6); 2 d (6); 4 d (6); 7 d (4); 11 d (6); 14 d (86); 21 d (40); 28 d (42); 35 d (4); 49 d (7); 63 d 5); 77 d (7); 91 d (8). The model is shown in Figure 1; the GM data and the model-calculated curve are also shown in Figure 3. FD_p_, fraction of dose in plasma,

GM, geometric mean.

**Supplemental WinSAAM Deck**

A SAAM31 GHANA FD N=87 June 4, 2024

H PAR

C Insert model parameters here

CC M(5) GMEAN 14d [ROHp]*Plasma volume

CC M(5) 3.362

IC(1) 1

L(2,1) 30

L(0,1) 9.99

L(3,2) 30

L(5,2) 0.3

L(4,3) 1

L(5,4) 9.451359E-01 2.857997E-01 2.572197E+00

L(6,5) 5.258398E+00 2.244237E+00 2.019813E+01

L(5,6) 1.843918E-02 7.430600E-03 6.687541E-02

L(7,5) 4.742802E+00 1.140363E+00 1.026327E+01

L(5,7) 2.969572E-01 6.146666E-02 5.532000E-01

L(9,6) 7.035746E-04 9.999998E-05 9.999998E-04

CC half of the total system output was set to come from irreversible tissue

CC utilization coming out of a delay (8) that does not recycle to plasma

L(8,5)= G(8)

XG(8)=R(9,6)/M(5)

L(9,8) 1

DT(3) 1.298699E-01 4.951100E-02 4.455990E-01

DT(8) 0.052

DN(3) 8

DN(8) 8

H STE

U(1) 1.732277E+00 100

M(5) 3.362

H DAT

C Insert data values here

CC output from “stores” was given some weight based on estimated diet VA

CC intake and absorption efficiency

100 FSD=0.075

R(9,6) 1 0.6525

CC TIME (d) FD

105 FSD=0.1

0 0

0.25 0.030796555

105 FSD=0.05

0.375 0.054393628

0.5 0.054295118

105 FSD=0.1

1 0.037900751

2 0.023527879

4 0.009552948

CC d7 AND d35 ARE THE AVERAGE OF ONLY 4 SUBJS so lower weight

105 FSD=0.5

7 0.004192439

105 FSD=0.1

11 0.004379172

105 FSD=0.02

14 0.003393966

105 FSD=0.05

21 0.002644428

28 0.002385991

105 FSD=0.5

35 0.003302764

105 FSD=0.1

49 0.002413200

63 0.002028369

77 0.002144149

91 0.002124889

CC SIMULATIONS TO SMOOTH CURVE

105

0.1

2 0.1 9

2 1 9

2 5 16

100

M(4)

M(5)

M(6)

M(7)

R(9,6)

R(8,5)

U(1)

CC PLASMA RETINOL SA as Fraction of Dose

CC SAp

125G(25)

XG(25)=F(5)/M(5)

1

2 1 95

CC TBS

129G(29)

XG(29)=M(6)+M(7)

1

CC FaS

CC Fa

136G(36)

XG(36)=(F(6)+F(7))

1

2 1 95

CC S

137G(37)

XG(37)=((F(5)/M(5))/((F(6)+F(7))/

(M(6)+M(7))))

1

2 1 95

CC FaS

135G(35)

XG(35)=G(36)*G(37)

1

2 1 95
